# Capacitance and Dielectric Properties of Spin-Coated Silk Fibroin Thin Films for Bioelectronic Capacitors

**DOI:** 10.3390/ma18071408

**Published:** 2025-03-22

**Authors:** Jongyun Choi, Seung Hun Lee, Taehun Kim, Kyungtaek Min, Sung-Nam Lee

**Affiliations:** 1 Department of IT Semiconductor Convergence Engineering, Tech University of Korea, Siheung 15073, Republic of Korea; 2 Department of Semiconductor Engineering, Tech University of Korea, Siheung 15073, Republic of Korea

**Keywords:** silk fibroin, spin coating, dielectric properties, bioelectronic capacitor, thin film, capacitance–voltage, dielectric loss

## Abstract

Silk fibroin, a biocompatible and flexible biopolymer derived from Bombyx mori silkworms, has shown promise in bioelectronics, due to its adjustable dielectric properties. This study investigates the influence of spin coating parameters on the optical, electrical, and dielectric properties of thin silk fibroin films. Silk fibroin solutions were spin coated onto indium tin oxide (ITO)/glass substrates at speeds ranging from 1000 to 7000 revolutions per minute (RPM), resulting in films with thicknesses that varied from 264.8 nm to 81.9 nm. Atomic force microscopy analysis revealed that the surface roughness remained consistent at approximately 1.5 nm across all the spin coating speeds, while the film thickness decreased with the increasing spin speed. Ultraviolet (UV)–visible spectroscopy showed that the transmittance at 550 nm increased from 81.2% at 1000 RPM to 93.8% at 7000 RPM, and the optical bandgap widened from 3.82 eV at 1000 RPM to 3.92 eV at 7000 RPM, which was attributed to reduced molecular packing and quantum confinement effects. Electrical characterization showed that thinner films (a spin speed of 5000–7000 RPM) exhibited a 15-fold increase in the leakage current, rising from 2.99 pA at 1000 RPM to 44.9 pA at 7000 RPM, and a decrease in resistance from 334 GΩ at 1000 RPM to 22.2 GΩ at 7000 RPM. The capacitance–voltage measurements indicated a 4-fold increase in voltage-dependent capacitance for thinner films, with capacitance values increasing from 36 pF at 1000 RPM to 176 pF at 7000 RPM. Dielectric loss analysis revealed that thinner films experienced higher energy dissipation at low frequencies (tan δ of 0.041 at 0.01 MHz for 7000 RPM), but lower losses at high frequencies (tan δ of 0.123 at 1 MHz for 7000 RPM). These findings emphasize the importance of film thickness control in optimizing the performance of silk fibroin-based bioelectronic devices.

## 1. Introduction

Silk fibroin, a naturally derived biopolymer, has gained significant attention in bioelectronics due to its biocompatibility, flexibility, and tunable dielectric properties [1,2,3]. Derived from silkworms (*Bombyx mori*), silk fibroin exhibits excellent mechanical strength, optical transparency, and controllable electrical characteristics, making it a promising material for applications such as flexible capacitors, optoelectronic devices, and bio-integrated circuits [4,5,6]. One of the key factors influencing its performance in electronic applications is the film thickness, which can be precisely controlled using spin coating [7,8]. This study explores the relationship between spin coating parameters, optical transmittance, capacitance behavior, and dielectric loss in thin silk fibroin films. Thin film capacitors are widely used in energy storage, sensing, and signal processing applications, where stable dielectric behavior is crucial [9,10,11]. The capacitance and leakage current of a dielectric material depends on its thickness, polarization response, and frequency-dependent behavior [12,13]. As the thickness of silk fibroin films decreases, the leakage current increases, resistance rises, and the voltage-dependent capacitance becomes more pronounced. These effects are particularly important in flexible electronics, where minimizing the dielectric loss and optimizing the frequency response are essential for stable operations. The optical properties of silk fibroin films also play a significant role in determining their suitability for optoelectronic devices. With an increasing spin coating speed, the film thickness decreases, leading to higher transmittance and a wider optical bandgap. Photoluminescence (PL) studies further reveal that thinner films exhibit a redshift and peak narrowing, suggesting changes in the molecular orientation and dipole interactions [14]. These optical characteristics make silk fibroin an excellent candidate for transparent electronic applications, including bio-sensors and light-sensitive capacitive devices [15,16].

In addition to optical and electrical properties, the C-V characteristics of silk fibroin capacitors exhibit quadratic function-like and asymmetric behavior, providing insights into their charge retention and polarization dynamics [17,18]. When a voltage is applied, charge carriers within the silk fibroin layer rearrange, and this rearrangement leads to a shift in the capacitance as the voltage is reversed. The observed asymmetry in the capacitance–voltage (C-V) curves becomes more pronounced as the film thickness decreases, suggesting that thinner films exhibit stronger polarization effects and slower dipolar relaxation [18]. This asymmetry and the quadratic nature of the C-V curve indicate the influence of surface and interface effects, which are more significant in regard to thinner films. Understanding this behavior is crucial for improving the performance and reliability of silk fibroin-based memory and capacitor devices. Furthermore, the frequency-dependent dielectric response of silk fibroin capacitors is crucial for assessing their performance in alternating current (AC) applications [19,20]. As the frequency increases, the capacitance decreases, due to dipolar relaxation limitations, with thinner films experiencing a sharper decline [21]. The dielectric loss tangent (tan δ) initially decreases at moderate frequencies, but rises at higher frequencies, due to conduction losses and interfacial polarization effects [22]. This frequency-dependent behavior underscores the importance of optimizing the film thickness to balance capacitance stability and minimize dielectric loss in bioelectronic circuits [23].

This study systematically examines the influence of spin coating parameters on the optical, electrical, and dielectric properties of thin silk fibroin films. Unlike previous research, which has primarily focused on the structural or mechanical properties of silk fibroin, this work provides a comprehensive analysis of its capacitance behavior, leakage characteristics, and frequency-dependent dielectric response. By precisely tuning the film thickness through spin coating, it is possible to control the transparency, capacitance variation, leakage current, and hysteresis behavior. The findings demonstrate that film thickness significantly impacts dielectric performance, with thinner films exhibiting stronger voltage-dependent capacitance and greater frequency-dependent losses. These insights contribute to the development of silk fibroin-based bioelectronic capacitors and flexible optoelectronic applications, offering a scalable and tunable approach to optimizing device performance.

## 2. Materials and Methods

**Figure 1:**
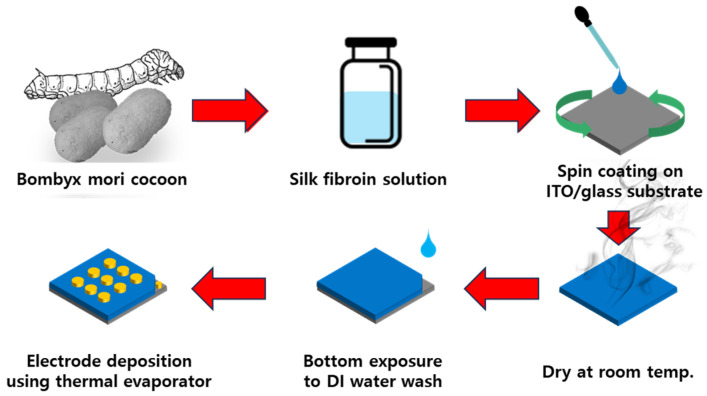
Schematic illustration of the fabrication process of a bioelectronic capacitor. Bombyx mori cocoon silk fibroin solution is prepared, spin coated onto an ITO/glass substrate, dried, and subsequently integrated with electrodes.

A silk fibroin solution was prepared following a standard protocol, as shown in Figure 1 [24]. First, a sodium carbonate (Na_2_CO_3_, Duksan LTD., Ansan, Republic of Korea) solution was heated, and shattered silkworm cocoons were immersed in it for 60 min to remove sericin. The degummed silk fibroin was then thoroughly rinsed with deionized (DI) water multiple times and, subsequently, dried. The dried silk fibroin was dissolved in a lithium bromide (LiBr, Sigma-Aldrich, Darmstadt, Germany) solution at a controlled temperature to ensure complete dissolution. The resulting solution was aged at 60 °C for 2 h to promote uniform fibroin dissolution. To purify the fibroin solution, a dialysis process was conducted using a dialysate membrane in DI water for 48 h, with periodic replacement of the water to remove LiBr and other impurities. Following dialysis, the solution was centrifuged at a suitable speed (4000 RPM) to remove undissolved residues. Finally, the purified silk fibroin solution was filtered, before its further use in the experiments. A silk fibroin-based bio-capacitor was fabricated using a solution derived from Bombyx mori silkworm cocoons. The cocoons were processed into an aqueous solution to obtain silk fibroin. ITO-coated glass substrates (iTASCO LTD, Seoul, Republic of Korea) were used as the base electrode. The prepared silk fibroin solution was deposited onto the ITO surface using a spin coating process. The spin coating speed varied from 1000 to 7000 RPM in 2000 RPM increments to control the film thickness. After coating, the samples were dried at room temperature for 10 min to allow solvent evaporation. To expose the bottom ITO electrode, the dried films were selectively etched using deionized water, ensuring the bottom surface was properly exposed. Finally, a 50 nm thick Al electrode was deposited on top of the silk fibroin layer using a thermal evaporator, completing the bio-capacitor fabrication.

The characteristics of the silk fibroin-based bio-capacitor were evaluated through the use of various analytical techniques. The surface morphology of the fabricated bio-capacitor was examined using optical microscopy (OM, Samwon, Seoul, Republic of Korea) to observe the macroscopic features and atomic force microscopy (AFM, Nanofocus, Seoul, Republic of Korea) was used for the high-resolution topographical analysis. Additionally, alpha-step profilometry was employed to measure the thickness and uniformity of the silk fibroin film. Ultraviolet (UV)–visible spectroscopy (Thermo Fisher Scientific, Evolution 300, Waltham, MA, USA) and photoluminescence (PL, Dongwoo, Seoul, Republic of Korea) measurements, using laser excitation, were performed to assess the optical properties. The electrical properties of the bio-capacitor were evaluated using a semiconductor parameter analyzer (HP 4155A, Santa Rosa, CA, USA) to measure current–voltage (I-V) characteristics. A precision LCR meter (Agilent 4284A, Santa Rosa, CA, USA) was utilized to analyze the capacitance and frequency-dependent impedance behavior of the device.

## 3. Results and Discussion

**Figure 2:**
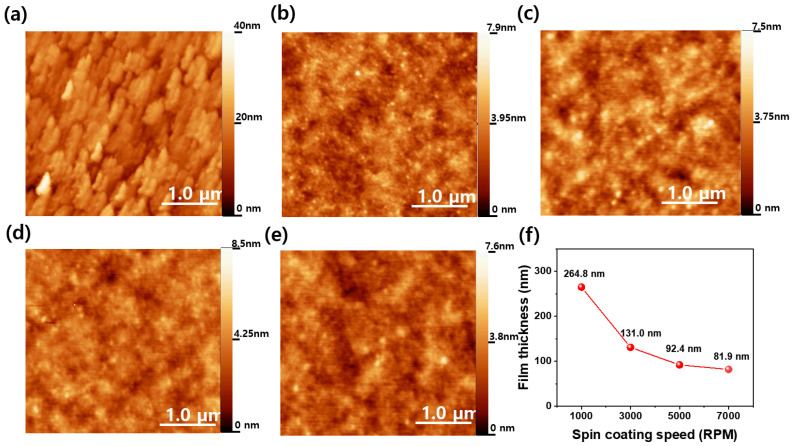
AFM image of (**a**) ITO/glass, and silk fibroin layer coated on ITO/glass substrates at spin coating speeds of (**b**) 1000, (**c**) 3000, (**d**) 5000, and (**e**) 7000 RPM. (**f**) Thickness of silk fibroin films on ITO/glass as a function of spin coating speed.

Figure 2a–e show the AFM images of the ITO/glass and silk fibroin layers on the ITO/glass substrates at spin coating speeds ranging from 1000 to 7000 RPM. The surface AFM images of the ITO/glass and the ITO/glass with a spin-coated silk fibroin layer exhibit significant differences in their morphology. The bare ITO/glass substrate exhibits a relatively high roughness of 4.31 nm, characterized by a textured surface, as shown in Figure 2a. In contrast, the silk fibroin films show a dramatic reduction in roughness across all the spin coating speeds, with values ranging from 0.75 nm to 0.77 nm. This suggests that spin-coated silk fibroin retains a consistent roughness regardless of the spin coating speed, effectively smoothing the surface, while maintaining uniformity in terms of shape. The roughness of thin silk fibroin films remains consistent when spin coated onto ITO/glass substrates, regardless of the spin coating speed. The viscosity and surface tension of the silk fibroin solution likely stabilizes the film formation process, thereby preventing significant variations in surface roughness across different spin speeds. Furthermore, the smoothness of the ITO/glass substrate, combined with the uniform distribution of the silk fibroin during spin coating, contributes to the maintenance of a consistent surface morphology. The graph of the film thickness versus the spin coating speed demonstrates the expected inverse relationship between the spin coating speed and the film thickness. At 1000 RPM, the film is the thickest at 264.8 nm. As the spin speed increases, the film thickness decreases significantly, reaching 131.0 nm at 3000 RPM, 92.4 nm at 5000 RPM, and, finally, 81.9 nm at 7000 RPM. This trend is consistent with the typical behavior of spin-coated films, where higher rotation speeds lead to thinner coatings, due to increased centrifugal force and solvent evaporation [25]. These results confirm that the spin coating method enables precise control over the silk fibroin film thickness, while achieving a smooth surface morphology, making it a suitable fabrication method for bioelectronic applications.

**Figure 3:**
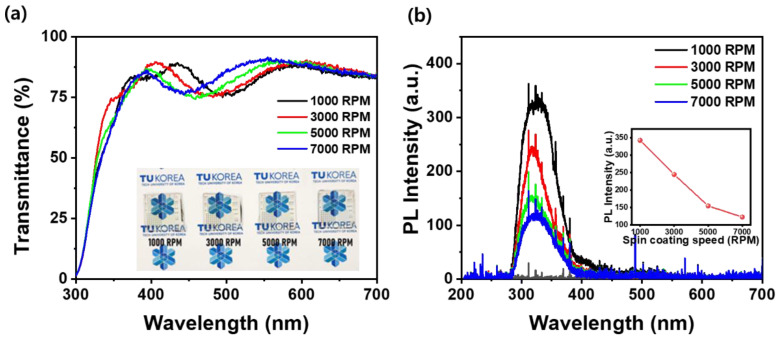
(**a**) Optical transmittance and (**b**) PL spectra of silk fibroin films deposited on ITO/glass substrates at spin coating speeds ranging from 1000 to 7000 RPM. The insets in (**a**,**b**) show photographic images of the Al/silk fibroin/ITO/glass structure and the PL intensity of silk fibroin films as a function of spin coating speed, respectively.

Figure 3a,b exhibit the optical transmittance measured using UV–visible spectroscopy and the PL measurement results, respectively, illustrating the optical properties of thin silk fibroin films spin coated onto ITO/glass substrates at different spin speeds (1000–7000 RPM). The optical transmittance spectra of the silk fibroin films indicate high optical transparency across the visible range, as shown in Figure 3a. Considering the change in transmittance from 300 nm to 700 nm due to variations in the film thickness, the thinnest sample deposited at 7000 RPM exhibits the highest transmittance (91.5%) at 550 nm compared to the other samples. In contrast, the relatively thicker films deposited at 1000 RPM and 3000 RPM show the highest transmittance at 400–450 nm, although still lower than the maximum transmittance at 550 nm observed for the 7000 RPM sample. While the thinnest silk fibroin films exhibit the highest transmittance, the observed oscillations, likely caused by Fabry–Perot interference, suggest that this difference may not be significant. The formation of these oscillations also indicates that the surface roughness of the films is very low, which aligns with the AFM results shown in Figure 2. Despite these oscillations, all the samples are sufficiently transparent to form transparent capacitors, as demonstrated by their relatively high transmittance in the inset of Figure 3a. The optical bandgap (E_g_) also exhibits a slight increase in the spin speed, from 3.82 eV at 1000 RPM to 3.92 eV at 7000 RPM, which can be attributed to reduced molecular packing and possible quantum confinement effects in regard to the thinner films [26,27]. As the film becomes thinner, the reduced intermolecular interactions lead to weaker electronic coupling between molecular orbitals, increasing the energy required for electronic transitions [26]. Additionally, quantum confinement effects become more pronounced at reduced thicknesses, restricting the charge carrier movement and further widening the bandgap [27]. These optical characteristics confirm the potential of silk fibroin films for transparent bioelectronic applications. Figure 3b exhibits the room-temperature PL spectra of silk fibroin films deposited on ITO/glass substrates at spin coating speeds ranging from 1000 to 7000 RPM. The PL intensity decreases as the spin coating speed increases, which correlates with the reduced film thickness. The most intense PL emission is observed at the lowest speed (1000 RPM), where the film is thickest, while the emission gradually weakens for thinner films, as shown in the inset of Figure 3b. Furthermore, the PL peak position exhibits a redshift from 327.6 nm at 1000 RPM to 323 nm at 7000 RPM, indicating changes in the molecular interactions, such as variations in hydrogen bonding and the molecular orientation within the film [28]. To ensure accurate determination of the peak position, Gaussian peak fitting was applied, minimizing the influence of spectral noise. While the observed redshift is relatively small, potential noise in the broad PL spectrum may introduce some uncertainty, and further refinement of spectral analysis methods could enhance accuracy in future studies. In addition, the full width at half maximum (FWHM) was measured to be 69 nm at 1000 RPM, decreasing to 54 nm at 3000 RPM, and increasing again to 64 nm at 7000 RPM. This variation in the FWHM is believed to be associated with oxygen-related defects or carbonyl-related states contributing to luminescence in the 330 nm region [28], which becomes more pronounced when the film is either too thick or too thin. Such emissions are typically attributed to structural defects, including oxygen-related defects and disorders in molecular packing, which influence the carrier recombination dynamics and broaden the emission spectrum [28].

In addition, as shown in Figure 3b, the blue emissions (400–450 nm) decreased as the spin coating speed increased, suggesting that the more aligned β-sheet structures formed at higher spin speeds led to reduced defect-related recombination. This alignment minimized the non-radiative recombination pathways, thereby enhancing the overall emission efficiency in the blue region [28]. This trend suggests that the luminescence properties of silk fibroin films can be tuned by controlling the spin coating parameters, which may be useful for optoelectronic and bio-imaging applications. Overall, these results highlight the influence of film thickness on the optical bandgap and PL characteristics of silk fibroin. The ability to control the transmittance, bandgap, and emission properties through spin speed adjustments demonstrates the potential of thin silk fibroin films for applications in flexible and transparent bioelectronic devices.

**Figure 4:**
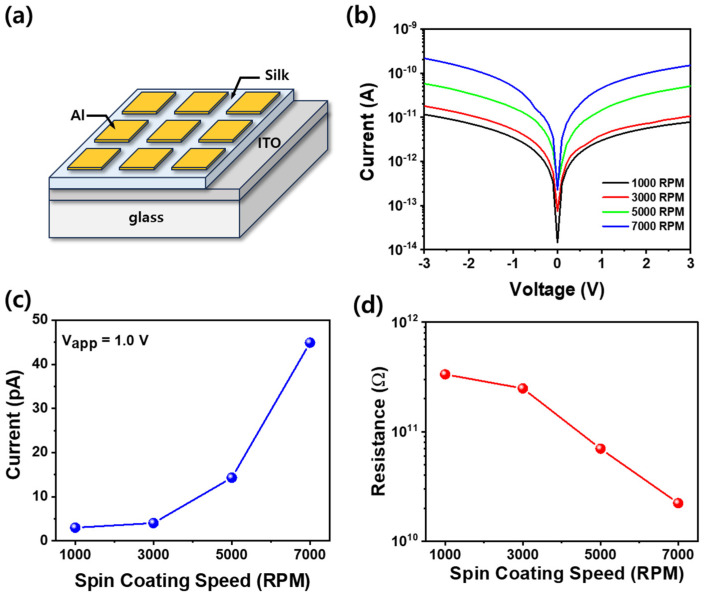
(**a**) Schematic diagram and (**b**) current–voltage curves of Al/silk fibroin/ITO capacitors at different spin coating speeds (1000–7000 RPM). (**c**) Leakage current and (**d**) resistance measured at 1.0 V of the Al/silk fibroin/ITO capacitors as a function of spin coating speed.

Figure 4a illustrates a schematic diagram of an Al/silk fibroin/ITO capacitor, where Al electrodes are deposited on a thin silk fibroin film spin coated onto an ITO/glass substrate. To measure the conductivity of the thin silk fibroin film, the current–voltage (I-V) characteristics of the capacitor, with an Al top electrode and an ITO bottom electrode, are presented in Figure 4b. The I-V characteristics of silk fibroin capacitors deposited at different spin coating speeds (1000–7000 RPM) reveal the dependence of the leakage current on film thickness. As the spin coating speed increases, the thickness of the thin silk fibroin film decreases, leading to an increase in the leakage current due to the shorter charge transport path and the enhanced electron tunneling effect. In particular, Figure 4c shows that the current measured at an operating voltage of 1.0 V exhibits a clear dependence on the spin coating speed. This trend is attributed to the reduction in the silk fibroin film thickness at higher spin speeds, which shortens the charge transport path and enhances electron tunneling. Specifically, the leakage current at 1.0 V increases from 2.99 pA at 1000 RPM to 44.9 pA at 7000 RPM, confirming the influence of the film thickness on the charge transport properties. Furthermore, as shown in Figure 4d, the resistance of the silk fibroin film measured at 1.0 V follows an inverse trend to the leakage current, as expected from Ohm’s law (R = V/I). The resistance of the silk fibroin capacitor is initially very high at 334 GΩ for a film coated at 1000 RPM, but decreases to 22.2 GΩ at 7000 RPM, as the film becomes thinner. This demonstrates that the thin silk fibroin film used in this study exhibits excellent dielectric properties, characterized by a low leakage current and high resistance. These findings emphasize the impact of film thickness on the electrical properties of silk fibroin, highlighting its potential for bioelectronic and flexible capacitor applications.

**Figure 5:**
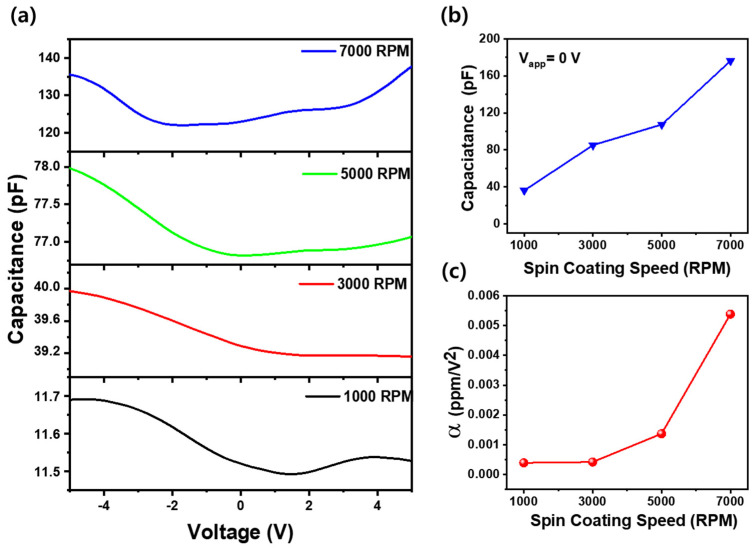
(**a**) C-V curves of Al/silk fibroin/ITO capacitors with different spin coating speeds (1000–7000 RPM). (**b**) Capacitance measured at an applied voltage of 0 V and (**c**) the voltage coefficient of capacitance of Al/silk fibroin/ITO capacitors as a function of spin coating speed of silk fibroin films.

The capacitance–voltage (C-V) characteristics of the Al/Silk/ITO capacitors fabricated at different spin coating speeds (1000–7000 RPM) were analyzed to investigate the dielectric properties of thin silk fibroin films. Figure 5a shows a parabolic C-V curve, which is one of the many shapes observed in regard to typical dielectric materials. This type of curve is often seen in materials with non-linear dielectric properties, such as silk fibroin films [29,30]. However, an asymmetry in the voltage-dependent capacitance is observed, likely due to charge-trapping effects or variations in the electrode interfaces. At 0 V, the initial capacitance values demonstrate a clear dependence on the spin coating speed, which directly affects the film thickness, as shown in Figure 5b. The capacitance increases as the spin speed increases, with values of 36 pF at 1000 RPM, 85 pF at 3000 RPM, 107 pF at 5000 RPM, and 176 pF at 7000 RPM. This trend aligns with the classical parallel plate capacitor equation, where capacitance is inversely proportional to film thickness. As the film becomes thinner with an increasing spin speed, the distance between the electrodes decreases, leading to an increase in capacitance. The voltage coefficient of capacitance (VCC, α), denoted as α = ΔC/(C_o_ΔV), was calculated to quantitatively assess the variation in capacitance according to the applied voltage, in addition to the capacitance at 0 V [31], as shown in Figure 5c. The results show that the VCC increases exponentially with the spin coating speed, or equivalently, as the film thickness decreases. Specifically, the VCC values are 3.93 × 10^−4^ ppmV^−2^ for 1000 RPM, 4.22 × 10^−4^ ppmV^−2^ for 3000 RPM, 1.38 × 10^−3^ ppmV^−2^ for 5000 RPM, and 5.38 × 10^−3^ ppmV^−2^ for 7000 RPM. This significant increase in the VCC for thinner films suggests stronger voltage-dependent capacitance behavior due to enhanced electric field effects. Thinner dielectric layers experience higher electric field strengths for the same applied voltage, leading to increased polarization and a non-linear dielectric response. Additional charge redistribution and interface states may contribute to the observed voltage-dependent capacitance variation. These results emphasize the importance of film thickness control in optimizing dielectric performance.

**Figure 6:**
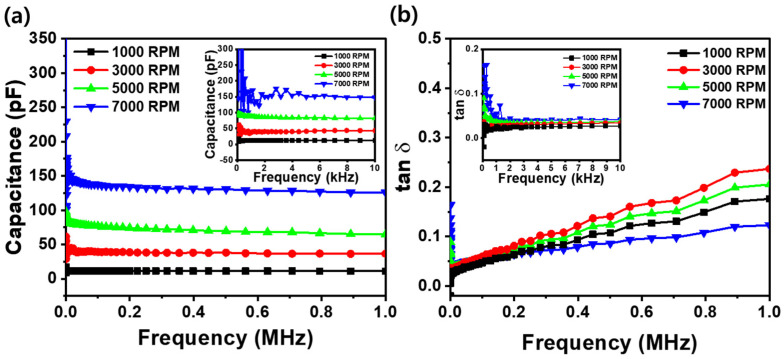
(**a**) Capacitance and (**b**) dielectric loss (tan δ) of Al/silk fibroin/ITO capacitors at different spin coating speeds (1000–7000 RPM) as a function of frequency.

Figure 6a,b illustrate the dielectric behavior of Al/Silk/ITO capacitors across different frequency ranges by presenting the measured capacitance and dielectric loss (tan δ), respectively. The results reveal distinct trends in the capacitance reduction, dependence on spin coating repetitions, and changes in the dielectric loss as the frequency increases. The capacitance measurements show a clear dependence on both film thickness and frequency, as shown in Figure 6a. As shown in the inset of Figure 6a, a silk fibroin film capacitor coated at 1000 RPM exhibits a capacitance of 10.8 pF at 0.2 kHz, whereas the capacitor coated at 7000 RPM shows a significantly higher capacitance of 229 pF, indicating the influence of film thickness on the dielectric properties. Although greater measurement variation occurs at low frequencies, particularly in regard to thinner films, the thicker film deposited at 1000 RPM still exhibits a lower capacitance, suggesting that film thickness plays a dominant role in determining the dielectric response despite the presence of measurement uncertainties. This trend is expected, as thinner films decrease the dielectric thickness, thereby increasing the electric field strength and enhancing the capacitance. This behavior aligns with the parallel-plate capacitor model, which describes capacitance as a function of two conductive plates separated by a dielectric material [32]. However, as the frequency increases to 1 MHz, the 1000 RPM sample maintains a capacitance of 11.8 pF, nearly unchanged from lower frequencies, while the 7000 RPM sample exhibits a significant decrease in capacitance to 125 pF, indicating a stronger frequency dependence in thinner films. As the spin coating speed of thin silk fibroin films decreases, the capacitance variation between low and high frequencies diminishes. In thicker films, such as those coated at 1000 RPM, the capacitance remains consistently low across all frequencies, indicating improved frequency stability. This behavior can be attributed to the restricted dipole alignment and charge storage capacity in thicker dielectric layers. In contrast, thinner silk fibroin films coated at 7000 RPM exhibit a higher capacitance at low frequencies, as the dipoles have sufficient time to align with the alternating electric field. However, at higher frequencies, the dipoles fail to respond quickly to the rapid field variations, resulting in a reduction in capacitance. Figure 6b illustrates the dielectric loss tangent (tan δ) as a function of frequency for thin silk fibroin film capacitors, with the spin coating speed varied from 1000 to 7000 RPM. The tan δ offers insight into the energy dissipation within the capacitor, representing the portion of energy lost as heat, due to the resistance of dielectric material to the applied electric field [33,34]. As shown in the inset of Figure 6b, at 1.0 kHz, capacitors with silk fibroin films coated at 1000 RPM exhibit a tan δ of 0.027, while thinner films coated at 7000 RPM show a higher value of 0.041. This is because thin silk fibroin film capacitors exhibit higher energy losses at low frequencies, where the dipoles have enough time to align with the electric field, but encounter increased resistance to reorientation, leading to a higher dielectric loss. In contrast, thicker silk fibroin films maintain lower tan δ values at low frequencies, as the dipoles in these films experience less resistance to alignment with the applied field, resulting in reduced energy loss.

As the frequency increases, the tan δ initially decreases for all the samples, reaching a minimum, before gradually increasing again at high frequencies. At 1.0 MHz, the tan δ value is 0.176 for the 1000 RPM silk fibroin capacitor, while the highest value of 0.237 is observed for the 3000 RPM silk fibroin capacitor. This increase in dielectric loss at 3000 RPM can be attributed to the optimal balance between dipole alignment and resistance to reorientation. At this spin coating speed, the dipoles align effectively align with the alternating electric field, leading to a higher dielectric loss. However, as the spin coating speed increases to 5000 and 7000 RPM, the film becomes thinner, which increases the resistance due to surface effects, defects, and increased barrier resistance at the interfaces. These factors hinder effective charge transport, resulting in a decrease in the tan δ to 0.123 at 7000 RPM. Consequently, the tan δ continuously decreases at higher spin coating speeds. Furthermore, the results in Figure 6a,b, which show different RPM-dependent trends in capacitance and the tan δ at 1.0 MHz for silk fibroin capacitors, indicate that the deviation can occur when the charge transport mechanism or interfacial effects become dominant. In particular, the deviation observed at 7000 RPM suggests a transition in regard to the charge transport behavior in ultrathin films, where the increased surface resistance and reduced dipole mobility influence the tan δ differently from the capacitance. This highlights the complex interplay between film thickness, polarization dynamics, and conduction-related losses, emphasizing the need to optimize the film thickness to achieve a balance between capacitance, frequency stability, and dielectric loss characteristics for bioelectronic applications.

## 4. Conclusions

This study investigated the optical, electrical, and dielectric properties of thin silk fibroin films fabricated via spin coating at different speeds (1000–7000 RPM). The results reveal significant insights into the impact of film thickness on the performance of silk fibroin, particularly in bioelectronic applications. The optical analysis showed that higher spin speeds produce thinner films, with increased transmittance and a widening optical bandgap. The PL measurements further indicated a redshift and a narrowing of emission peaks in thinner films, suggesting changes in the molecular interactions within thinner films. The electrical characterization of Al/Silk/ITO capacitors demonstrated that thinner films exhibit an increased leakage current, while the resistance and voltage-dependent capacitance exponentially rise as the film thickness decreases. The frequency-dependent capacitance measurements revealed that thinner films exhibit sharper capacitance decay at higher frequencies. Additionally, the dielectric loss increases due to conduction-related losses at low frequencies. These findings emphasize the importance of optimizing the film thickness and spin coating conditions to achieve stable performance in flexible capacitors, optoelectronic devices, and bio-integrated circuits. By enabling precise control over the dielectric properties of silk fibroin films, this study highlights their potential for a wide range of bioelectronic applications.

## Data Availability

The data presented in this study are available on request from the corresponding author. The data are not publicly available due to privacy concerns.
